# Clinical Relevance of Secreted Small Noncoding RNAs in an Embryo Implantation Potential Prediction at Morula and Blastocyst Development Stages

**DOI:** 10.3390/life11121328

**Published:** 2021-12-01

**Authors:** Angelika V. Timofeeva, Ivan S. Fedorov, Maria A. Shamina, Vitaliy V. Chagovets, Nataliya P. Makarova, Elena A. Kalinina, Tatiana A. Nazarenko, Gennady T. Sukhikh

**Affiliations:** 1 Laboratory of Applied Transcriptomics, Kulakov National Medical Research Center of Obstetrics, Gynecology and Perinatology, Ministry of Health of Russia, 117997 Moscow, Russia; is_fedorov@oparina4.ru; 2 Department of Assisted Reproductive Technologies, Kulakov National Medical Research Center of Obstetrics, Gynecology and Perinatology, Ministry of Health of Russia, 117997 Moscow, Russia; mariashamina@mail.ru (M.A.S.); np_makarova@oparina4.ru (N.P.M.); e_kalinina@oparina4.ru (E.A.K.); 3 Laboratory of Proteomics and Metabolomics of Human Reproduction, Kulakov National Medical Research Center of Obstetrics, Gynecology and Perinatology, Ministry of Health of Russia, 117997 Moscow, Russia; v_chagovets@oparina4.ru; 4 Kulakov National Medical Research Center of Obstetrics, Gynecology and Perinatology, Ministry of Health of Russia, 117997 Moscow, Russia; t_nazarenko@oparina4.ru (T.A.N.); g_sukhikh@oparina4.ru (G.T.S.)

**Keywords:** miRNA, piRNA, deep sequencing, qRT-PCR, morula, blastocyst, logistic regression model, sncRNA gene-target

## Abstract

Despite the improvements in biotechnological approaches and the selection of controlled ovarian hyperstimulation protocols, the resulting pregnancy rate from in vitro fertilization (IVF) protocols still does not exceed 30–40%. In this connection, there is an acute question of the development of a non-invasive, sensitive, and specific method for assessing the implantation potential of an embryo. A total of 110 subfertile couples were included in the study to undergo the IVF/ICSI program. Obtained embryos for transfer into the uterine cavity of patient cohort 1 (*n* = 60) and cohort 2 (*n* = 50) were excellent/good-quality blastocysts, and small noncoding RNA (sncRNA) content in the corresponding spent culture medium samples at the morula stage (*n* = 43) or at the blastocyst stage (*n* = 31) was analyzed by deep sequencing followed by qRT-PCR in real time. Two logistic regression models were developed to predict the implantation potential of the embryo with 100% sensitivity and 100% specificity: model 1 at the morula stage, using various combinations of hsa_piR_022258, hsa-let-7i-5p, hsa_piR_000765, hsa_piR_015249, hsa_piR_019122, and hsa_piR_008112, and model 2 at the blastocyst stage, using various combinations of hsa_piR_020497, hsa_piR_008113, hsa-miR-381-3p, hsa_piR_022258, and hsa-let-7a-5p. Protein products of sncRNA potential target genes participate in the selective turnover of proteins through the ubiquitination system and in the organization of the various cell cytoskeleton and nucleoskeleton structures, regulating the activity of the Hippo signaling pathway, which determines the fate specification of the blastomers.

## 1. Introduction

In recent decades, different approaches to improve assisted reproductive technology (ART) have been developed. These include the selection of controlled ovarian hyperstimulation (COH) protocols and luteal phase support [1,2]; the use of intracytoplasmic sperm injection (ICSI) to avoid the influence of the male factor on fertilization [3]; adoption of oocyte and embryo vitrification techniques, with preservation of morphological characteristics after defrosting [4]; the development of time-lapse imaging to follow embryo development over time and to predict embryo viability [5,6,7]; the introduction of preimplantation genetic diagnosis (PGD) to reveal chromosomal aneuploidy [8,9].

Despite the improvements in biotechnological approaches, the resulting pregnancy rate from in vitro fertilization (IVF) protocols still does not exceed 30–40% [10]. According to the results of numerous studies, the selection of embryos for transfer into the uterine cavity only using morphological criteria has a relatively weak correlation with implantation [11]. Moreover, it was concluded that the parameters used for conventional blastocyst evaluation (morphology and developmental rate) were not adequate indicators to improve the selection, even among euploid embryos. To solve this problem, an attempt was made to combine conventional morphokinetic parameters (the time of the second polar body emission; the time of appearance of the two pronuclei; the time of their fade-out; the division time to 2–8 cells; the time from ICSI to early compaction; the time of morula formation; the time to early blastulation; the time to full blastocyst; the time to expanded blastocyst; and the time to early hatching blastocyst) and novel morphodynamic parameters (distance and speed of pronuclear migration, blastocyst expanded diameter, inner cell mass area, and trophectoderm cell cycle length), while developing an artificial neural network (ANN) model [12]. It was found that the new parameters analyzed were responsible for the increase in the predictive power for implantation, but the accuracy of the method did not exceed 0.76. Moreover, the lag in the rate of development of the embryo does not always determine the lack of implantation potential. It was revealed that the clinical pregnancy rate per embryo transfer was comparable between the blastocysts that developed from a morula on day 5 and blastocysts that developed from cavitating morulae on day 5 (21.3% vs. 24.7%) [13].

A further means of assessing embryo viability is to measure its metabolic activity [14,15,16]. A lower metabolic turnover has been reported in viable developing embryos, whereas a struggling nonviable embryo has been shown to exhibit a high rate of metabolism as it seeks to repair and survive [14]. Blastocysts that lack the ability to adhere in vitro had an increased requirement of pyruvate and lactate and were accompanied by a significant reduction of the pyruvate-alanine ratio in the culture medium [16]. Culture medium analysis by mass spectrometry identified implanting and nonimplanting embryos with 100 and 70% accuracy, respectively [17]. However, live birth rate was not significantly different in the group in which embryos were selected on metabolic profile of the culture media using near infrared spectroscopy, compared to morphology only in a randomized controlled trial [18]. The combination of proteomic techniques used to investigate the secretome of the spent culture media and the morphokinetic parameters of the embryos was used as a prognostic tool to predict implantation rates [19]. However, one of the main limitations of this study was the low sensitivity of the immunoassay technique used to detect IL-6, IFN-α2, and SCF in the spent culture media.

In connection with the above, there is an acute question of the development of a non-invasive, sensitive, and specific method for assessing the implantation potential of an embryo, with the aim of improving the clinical outcomes in ART. In this context, sncRNAs, which are the main regulators of embryo genome activity at transcriptional and posttranscriptional levels and are secreted by the growing embryo into the spent culture medium in free or extracellular vesicle (EV)-bound forms, can reflect development parameters of an embryo and its implantation potential [20,21,22,23]. MicroRNAs have been widely associated with mammalian development, particularly during the reprogramming of the transcriptome and epigenome. The literature has shown that miRNAs may target hundreds of critical genes, whose expression or repression determines the fate of the embryo [24]. However, lack of reproducibility in the detection of miRNAs in a spent culture medium questioned the use of miRNAs as a reliable biomarker and sharply raised the question of the need to search for new markers of implantation among other molecules among sncRNAs. In our previous studies, we showed the prevalence of piRNA molecules over miRNAs in the spent culture media during early embryogenesis and found markers associated with different rates of embryo development by the fourth day a.f. [25] and with different developmental outcomes of morula by the fifth day (degeneration or developmental arrest, a blastocyst of poor quality, or a blastocyst of excellent quality) [26].

In the present study, we aimed to develop models to prognose the implantation potential of an embryo at morula and blastocyst development stages based on miRNA and piRNA content in spent culture medium on Day 4 a.f. and Day 5 a.f., respectively. In combination with morphokinetic, CCS, metabolomic, and proteomic data, these models will potentially be helpful in improving selection of the single embryo with the best implantation potential.

## 2. Materials and Methods

### 2.1. Clinical Characteristics of Couples

In the current study, 110 subfertile couples were included, who received medical treatment in the Department of Assisted Technologies in the Treatment of Infertility named after Professor B.V. Leonov of the FSBI «National medical research center for obstetrics, gynecology and perinatology named after academic V.I. Kulakov». Couples with contraindications for the IVF/ICSI program were not included in the study (https://docs.cntd.ru/document/902369756, last accessed on 1 February 2021).

The patients included in the study underwent peripheral blood hormonal analysis (prolactin, 17-OH-progesterone, dehydroepiandrosterone sulphate, cortisol, testosterone, anti-müllerian hormone, thyroid-stimulating hormone, free thyroxine, luteinizing hormone, progesterone, follicle-stimulating hormone, and estradiol) in the Clinical Diagnostic Laboratory of the FSBI «National medical research center for obstetrics, gynecology and perinatology named after academician V.I. Kulakov» no earlier than six months before the date of entry into the ART program.

Married couples underwent an IVF program according to the standard protocol for stimulating ovarian function from 2–4 days of the menstrual cycle with gonadotropin-releasing hormone antagonists (antGnRH) and gonadotropins (https://docs.cntd.ru/document/902369756, paragraph 24 last accessed on 1 February 2021). The average age of women included in the study was 32.4 years (23–47 years). The average age of men was 34.8 years (25–56 years). A total of 52 married couples were diagnosed with primary infertility, and 58 married couples with secondary infertility. Twenty-eight out of 110 women underwent unilateral salpingectomy, and 14 out of 110 women underwent bilateral salpingectomy. According to the spermogram analysis, which was performed on the day of transvaginal ovarian puncture, 15 patients were found to have normozoospermia; 52, teratozoospermia; 11, oligoteratozoospermia; 9, oligoasthenoteratozoospermia; 11, asthenoteratozoospermia; 11, asthenozoospermia; and 1, cryptozoospermia. An isolated tubo-peritoneal factor of infertility was identified in 9 married couples, an isolated male factor of infertility was identified in 62 married couples, and a combined factor of infertility was identified in 33 married couples. The average duration of infertility among married couples was 4.4 years (1–10 years). A total of 63 married couples did not have any previous IVF attempts, 24 married couples had 1 IVF attempt, 15 married couples had 2 IVF attempts, and 8 married couples had more than 2 IVF attempts.

The average dose of gonadotropins in the patients included in the study was 1661 UI (525–3975 UI), and ovarian stimulation lasted approximately 9 days (6–19 days).

Immediately after follicular fluid aspiration during oocyte retrieval, the number of oocyte–cumulus complexes (OCCs) and the maturity of the retrieved oocytes were identified under a stereomicroscope on the heated surface of a sterile laminar box. A stable temperature (37.0 °C) was constantly maintained during all manipulations. For pre-incubation, all OCCs were washed from follicular fluid and blood and placed in sterile plates (Thermo Fisher Scientific Nunc A/S, Roskilde, Denmark) with Continuous Single Culture medium (CSCM, Irvine Sc., Santa Ana, CA, USA) for 2–3 h at a temperature of 37.0 °C and with 6% CO_2_. After the pre-incubation period, the oocytes were denuded, and cumulus cells that surrounded the oocytes were removed using hyaluronidase solution (Irvine Sc., Santa Ana, CA, USA). The oocytes were placed in hyaluronidase solution for 2 min.

Then, OCCs were washed again in the CSCM and returned to the wells. Retrieved oocytes at metaphase II stage were fertilized by the ICSI method and then transferred back to the CSCM for further cultivation.

The appearance of two pronuclei was observed 14–16 h after fertilization. If the presence of two pronuclei in the oocyte was not visualized at that point, the fertilization was considered failed. All embryos were cultivated in multigas incubators, produced by COOK (Australia, Brisbane), in 25 μL drops with oil (Fujifilm, Irvine Sc., Santa Ana, CA, USA).

In 60 married couples (1 cohort), on day 4 after fertilization, a 25 μL aliquot of spent culture medium was collected from each embryo at the morula stage, according to Tao J. et al. [27], placed into individual sterile tubes (SSI, Lodi, CA, USA) and subsequently frozen in liquid nitrogen and stored at −70 °C. Each morula was transferred into 25 μL of fresh CSCM-C medium for further cultivation until the 5th day after fertilization.

In 50 married couples (cohort 2), on day 5 after fertilization, a 25 μL aliquot of spent culture medium was collected from each embryo at the blastocyst stage, according to the Gardner grading scale [28,29], placed into individual sterile tubes (SSI, Lodi, CA, USA), and subsequently frozen in liquid nitrogen and stored at −70 °C.

The embryos for transfer into the uterine cavity of patients in cohorts 1 and 2 were excellent/good-quality blastocysts, and the corresponding spent culture medium samples at the morula stage (cohort 1) or at the blastocyst stage (cohort 2) were analyzed by deep sequencing and/or qPCR.

On the 14th day after embryo transfer, patients in cohorts 1 and 2 underwent b-HCG testing to diagnose pregnancy. In the case of a positive b-HCG result (>35 IU/L), the patients underwent transvaginal ultrasound to confirm the onset of a clinical intrauterine pregnancy. Of the 110 patients enrolled in the study, the onset of clinical pregnancy was diagnosed in 32 cases, including 6 cases of the missed miscarriage, 1 case of ectopic pregnancy, and 25 cases of healthy births.

All spent culture medium samples were collected between 2018 and 2020. The negative outcome of the ART program was the absence of a full-term fetus (no implantation, a biochemical pregnancy, an ectopic pregnancy or a missed miscarriage). The positive outcome of the ART program was the birth of a healthy, full-term fetus.

### 2.2. Experimental Design

**Figure 1:**
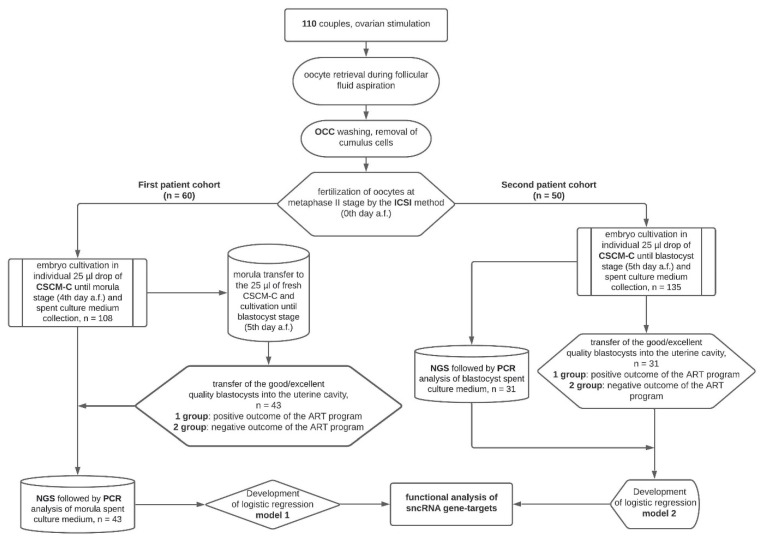
Flow diagram of the experimental design.

The research consisted of three main stages: (1) embryological stage, with the formation of embryo groups at morula and blastocyst development stages with different implantation potential; (2) quantitative analysis of sncRNA in morula groups by deep sequencing and qPCR in spent culture medium to develop logistic regression models (1—for the morula, 2—for the blastocyst) to prognose implantation potential; (3) functional analysis of target genes of sncRNAs associated with implantation potential at different development stages—morula and blastocyst (Figure 1).

### 2.3. Extraction of RNA from Spent Culture Medium

Twenty-five microliters of embryo culture medium adjusted to 200 μL with 0.9% NaCl were treated with 1000 µL of QIAzol Lysis Reagent (Qiagen, Hilden, Germany), followed by mixing with 200 µL of chloroform, centrifugation for 15 min at 12,000× *g* (4 °C), collection of 600 µL aqueous phase, and RNA isolation using the miRNeasy Serum/Plasma Kit (Qiagen, Hilden, Germany).

### 2.4. cDNA Library Preparation and RNA Deep Sequencing

cDNA libraries were synthesized using 7 µL of the 14 µL total RNA column eluate (miRNeasy Serum/Plasma Kit, Qiagen, Hilden, Germany), extracted from spent culture medium using the NEBNext^®^ Multiplex Small RNA Library Prep Set for Illumina^®^ (Set1 and Set2, New England Biolab^®^, Frankfurt am Main, Germany), amplified for 30 PCR cycles, and sequenced on the NextSeq 500 platform (Illumina, San Diego, CA, USA). The adapters were removed using Cutadapt. All trimmed reads shorter than 16 bp and longer than 50 bp were filtered out. The remaining reads were mapped to the GRCh38.p15 human genomes, miRBase v21, and piRNABase with bowtie aligner [30]. Aligned reads were counted using the featureCount tool from the Subread package [31] and with the fracOverlap 0.9 option, so the whole read was forced to have 90% intersection with sncRNA features. Differential expression analysis of the sncRNA count data was performed with the DESeq2 package [32].

### 2.5. Quantitative Real-Time RT-PCR

Five microliters of the 14 µL total RNA column eluate (miRNeasy Serum/Plasma Kit, Qiagen, Hilden, Germany) extracted from the embryo culture medium was converted into cDNA in a reaction mixture (20 µL) containing 1× HiSpec buffer, 1× Nucleics mix, and miScript RT, according to the miScript^®^ II RT Kit protocol (Qiagen, Hilden, Germany); then, the sample volume was adjusted with deionized water to 200 µL. The synthesized cDNA (2 µL) was used as a template for qPCR using a forward primer specific to the studied sncRNA (Table 1) and the miScript SYBR Green PCR Kit (Qiagen, Hilden, Germany). The following qPCR conditions were used: (1) 15 min at 95 °C and (2) 50 cycles at 94 °C for 15 s, an optimized annealing temperature (45–61.6 °C) for 30 s, and 70 °C for 30 s; followed by heating the reaction mixture from 65 to 95 °C in 0.1 °C increments to plot the melting curve of the qPCR product in a StepOnePlus™ thermocycler (Applied Biosystems, Foster City, CA, USA). The relative expression of sncRNA in the embryo culture medium was determined using the ∆∆Ct method, using has_piR_020388 (DQ598008, piRNAbank) as the reference RNA and culture medium, without contact with any embryo incubated for 4 or 5 days at 37 °C as a reference sample to calculate the fold change of expression level in a sample.

### 2.6. Statistical Analysis of the Obtained Data

For statistical processing of the results, we used scripts written in the R language [31] and RStudio [33]. The correspondence of the analyzed parameters to the normal distribution law was assessed using the Shapiro–Wilk test. When the distribution of data was different from normal, the Mann–Whitney test was used for paired comparison, and data were described as median (Me) and quartiles Q1 and Q3 in the Me format (Q1; Q3). To identify the relationship between categorical variables, chi-square testing was performed. The value of the threshold significance level *p* was taken as equal to 0.05. If the *p*-value was less than 0.001, then it was indicated in the format *p* < 0.001.

A logistic regression method was used to analyze the relationship between the sncRNA levels and the probability of embryo implantation. To select the optimal predictive model, a step-by-step exclusion of the predictor algorithm was used, with an estimation of the quality change based on the maximum likelihood function and the number of predictors. The probability values calculated based on the model were associated with the a priori data for the group without implantation and the group with implantation, taking into account the optimal ratio of sensitivity and specificity.

## 3. Results

### 3.1. Comparative Analysis of Clinical Data and Parameters of the IVF/ICSI Protocol in Couples, Depending on the Outcome of the ART Program

Anamnestic data and clinical and hormonal parameters were analyzed in the patients included in the study (Table 2). There were no statistically significant differences in the clinical and anamnestic characteristics of the patients in the study groups, except for the levels of testosterone, luteinizing hormone (LH), and anti-müllerian hormone (AMH). Although these three hormone levels fell within the range of reference values, AMH levels were reduced (*p* = 0.007737) and LH and testosterone levels were elevated (*p* = 0.036986 and *p* = 0.017549, respectively) in the women with a negative outcome of the ART program versus the women with a positive outcome of the ART program (delivery of healthy fetus). The above statistically significant changes in the hormonal profile along with a trend toward lower estradiol levels (154.5 pmole/L vs. 165.75 pmole/L, *p* = 0.32861) and higher follicle-stimulating hormone levels (7.55 IU/L vs. 6.41 IU/L, *p* = 0.094989) may be indicative of premature ovarian insufficiency [34], proposed to be in a subclinical form in the group of patients with a negative outcome of the ART program.

**Figure 2:**
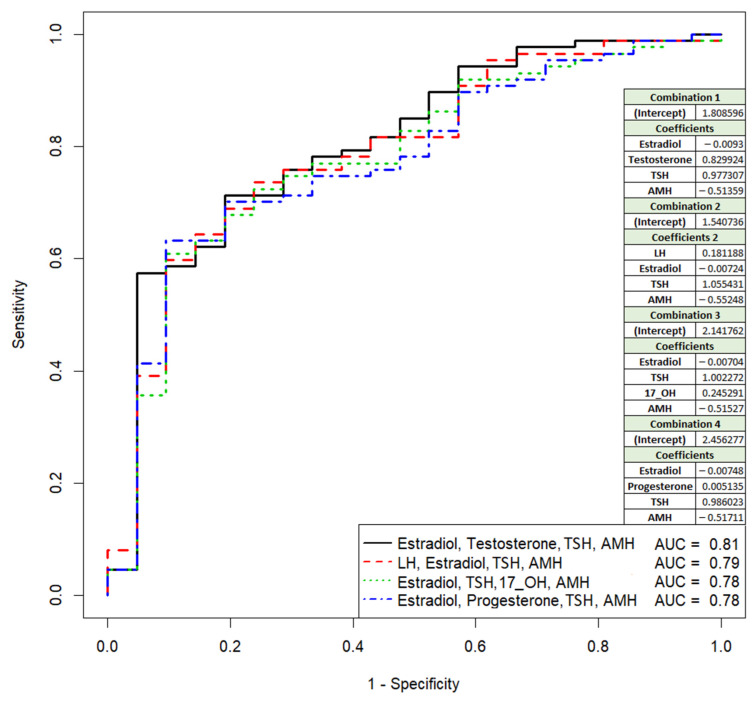
Receiver operating characteristic (ROC) curves of the logistic regression models to predict pregnancy based on hormonal data for all women included in the study. TSH—thyroid-stimulating hormone, AMH—anti-müllerian hormone, 17_OH—17-OH-progesterone.

The insufficient sensitivity, specificity, and accuracy to predict the pregnancy based on hormonal data for all women included in the present study (Table 2) before beginning the ART program is demonstrated in Figure 2 and Table S1 (Sheet 1). For instance, AUC = 0.807 (*p* < 0.01), accuracy = 64.8%, sensitivity = 57.5%, specificity = 95.2%, and true positive rate = 57.4% are for the model developed based on the combination of the levels of estradiol, testosterone, TSH, and AMH, while the combination of LH, estradiol, TSH, and AMH results in the following model parameters: AUC = 0.79 (*p* < 0.01), accuracy = 65.7, sensitivity = 59.8, specificity = 90.5, and true positive rate = 59.8%. Thus, the focus of our attention was the identification of more sensitive and specific prognostic markers of the pregnancy onset at the embryological stage as a part of the ART program.

The general formula of the developed model variants is as follows:

(1) $$e=\frac{1}{1+{\;e}^{-i-к1*x1-к2*x2-\ldots }}$$

where i—intercept term; k1, k2,…—coefficients for each of the hormones; x1, x2,… —levels of the corresponding hormones.

This model could be useful for a preliminary assessment of a woman’s fertility according to her hormonal profile before entering an ART program.

### 3.2. Identification of sncRNAs Associated with Implantation Potential of Embryo at Morula Stage

To search for sncRNAs determining the implantation potential of the morula on day 4 after fertilization, we chose samples of spent culture medium from the morula that had developed into blastocysts of good/excellent quality on day 5 after fertilization from couples of cohort 1 (*n* = 43 among all obtained 108 blastocysts of different quality). Of these, after their transfer into the uterine cavity, 10 blastocysts were implanted, and 33 blastocysts were not implanted.

#### 3.2.1. Deep Sequencing of the Spent Culture Medium

Deep sequencing was performed on seven samples of spent culture media from the morula on day 4, developed into blastocysts nonimplanted (4 of 33 embryos) and implanted (3 of 10 embryos) after transfer into the uterine cavity, and on one sample of culture medium without embryo. Sequence reads aligned to miRBase v21 and piRNABase, with a count of at least five in one sample studied, were normalized in DeSeq2 package and compared in two formed groups (with and without implantation). The results of the comparison in the form of the statistical significance of these differences are presented in Table 3.

#### 3.2.2. Quantitative RT-PCR Analysis in Real Time

The expression level of sncRNAs indicated in Table 3 was quantified by real-time RT-PCR in all collected spent culture medium samples from the morula, developed into blastocysts of good/excellent quality, with different outcomes after transfer to the uterine cavity (*n* = 43). Of these, hsa_piR_020388 and hsa_piR_020485 were also taken for analysis as molecules for normalizing RT-PCR data. Specifically, hsa_piR_020388 was chosen as the reference RNA due to its consistent expression level in all analyzed samples.

**Figure 3:**
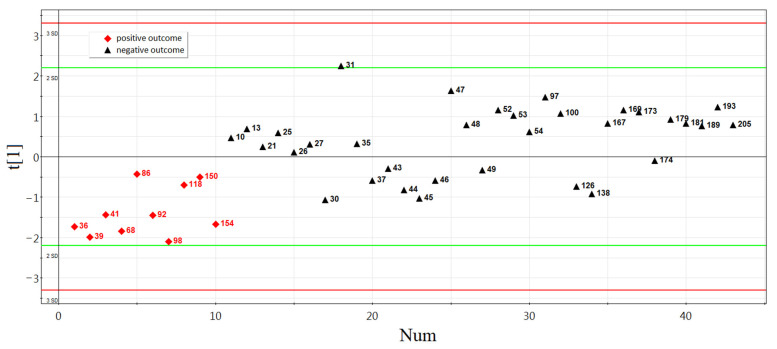
Principal component analysis (PCA) plot based on the sncRNA dataset for the morula stage.

The principal component analysis was used to determine the clustering of studied samples based on the RT-PCR data (Figure 3). The logarithm to base two of fold change expression level of sncRNA in each of the spent culture medium samples relative to the culture medium without embryo, calculated as a (−ΔΔCt) value, was used in this analysis (−ΔΔCt data are presented in Table S1, Sheet 2). Figure 3 shows that samples of the culture medium from morulae, followed by implantation (indicated by red squares), form a separate cluster and differ in the expression profile of small noncoding RNAs from the significant majority (79%) of the culture medium samples from morulae that were not implanted after they reached the blastocyst stage and transferred into the uterine cavity (indicated by black triangles). The absence of embryo implantation from patients with ID numbers 30, 37, 44, 45, 46, 126, and 138, despite the similarity of the sncRNA expression profile in the morula spent culture medium with that in the spent culture medium samples from morula with implantation potential, may not be associated with the molecular biological characteristics of the embryo itself, but may be due to the lack of endometrium receptivity at the time of embryo transfer into the uterine cavity and/or chronic endometritis associated with adnexitis. For example, patient #30 had a history of primary infertility for 2 years and bilateral salpingectomy; patient #37—secondary infertility for 4 years and bilateral salpingectomy; patients #44 and #46—primary infertility for 5 and 7 years, respectively; patient #45 had secondary infertility for 3 years and unilateral salpingectomy; patient #126 had secondary infertility for 5 years and unilateral salpingectomy. Therefore, these samples, representing 21% of all samples in the non-pregnant group, were excluded from further analysis.

**Figure 4:**
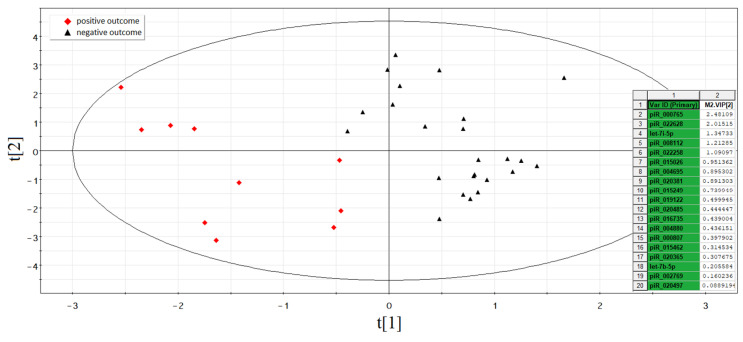
PLS-DA model for morula stage.

According to the Partial Least Squares Discriminant Analysis (PLS-DA) model based on RT-PCR data without outlier samples, two clusters of data points can be clearly distinguished (Figure 4). The first one (highlighted in red) has an abscissa of less than −0.4 and represents the samples of spent culture medium from the morula developed into good/excellent blastocysts and resulting in normal pregnancy after their transfer to the uterine cavity. The second cluster (highlighted in black) has an abscissa of more than −0.4 and represents the samples of spent culture medium from the morula developed into good/excellent blastocysts with no implantation.

The contribution of sncRNAs to the distribution of the data points on the score plot of the developed PLS-DA model was estimated by the Variable Importance in Projection (VIP) values for groups of embryos with and without implantation. The VIP values are presented as an insert in the right side of the score plot. The sncRNAs with VIP ≥ 1 and the highest impact for the differentiation of implanted and nonimplanted embryos were as follows: hsa_piR_000765 (VIP = 2.48), hsa_piR_022628 (VIP = 2.02), hsa-let-7i-5p (VIP = 1.35), hsa_piR_008112 (VIP = 1.21), hsa_piR_022258 (VIP = 1.09), and hsa_piR_015026 (VIP = 0.95).

#### 3.2.3. Development of Logistic Regression Model 1

**Figure 5:**
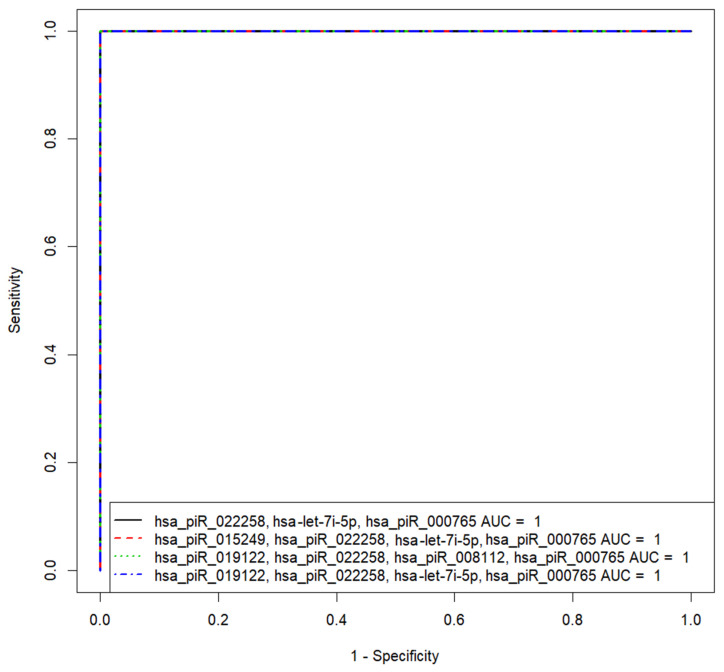
Receiver operating characteristic (ROC) curves of the logistic regression models to predict implantation potential of embryo at the morula stage.

Based on the (−ΔΔCt) values of sncRNA in the spent culture medium from the morula of couples with negative and positive results of the ART program, different variants of logistic regression model 1 for calculating the probability of the embryo implantation were developed (Figure 5). Receiver operating characteristic (ROC) curves of the developed logistic regression model 1 variants are presented in Table 4.

The general formula of model 1 variants is (1) as mentioned above, but where i—intercept term; k1, k2,…—coefficients for each of the sncRNAs; x1, x2,…—sncRNA “−ΔΔCt” values.

We found that the diagnostic accuracy of expression profiling of any combination of sncRNAs was 100% (AUC = 1, sensitivity—100%, specificity—100%, true positive rate—1; false positive rate—0).

### 3.3. Verification of the Predictive Value of sncRNA Markers Associated with Implantation Potential of Morula at the Blastocyst Stage

**Figure 6:**
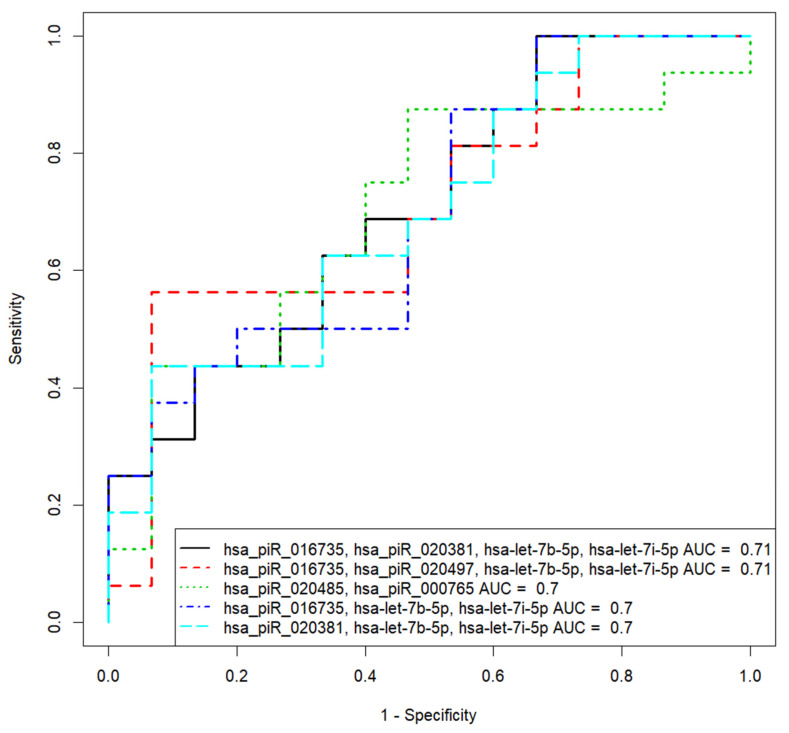
Receiver operating characteristic (ROC) curves of the logistic regression models to predict the implantation potential of embryo at the blastocyst stage based on the expression level of sncRNAs associated with implantation potential of embryo at the morula stage.

From 135 spent culture media samples from blastocysts of different quality obtained from 50 couples in cohort 2 (Figure 1), 31 spent culture media samples from blastocysts of good/excellent quality with different implantation potential after transfer into the uterine cavity (with no implantation—16, with implantation—15) were selected. In the selected samples, the expression level of those sncRNAs that participated in the development of the PLS-DA model 1 for the morula stage was analyzed (the list of molecules is presented in the inset of Figure 3). PiRNA hsa_piR_020388 was chosen as the reference RNA due to its consistent expression level in all analyzed samples. Based on the (−ΔΔCt) values of sncRNA in the spent culture medium from blastocysts of couples with negative and positive results of the ART program (−ΔΔCt data are presented in Table S1, Sheet 3), different variants of the logistic regression model for calculating the probability of the embryo implantation were developed (Figure 6; Table S1, Sheet 4). We found that the diagnostic accuracy of expression profiling of the different combinations of sncRNAs presented in Figure 6 was less than 100%, among which the best combination was hsa_piR_016735, hsa_piR_020381, hsa-let-7b-5p, and hsa-let-7i-5p: AUC = 0.708, *p* = 0.025; accuracy—67.7%, sensitivity—100%, specificity—33.3%, true positive rate—1; false positive rate—0.66.

In this regard, we aimed to find other molecules whose expression profile at the blastocyst stage would accurately predict the onset of pregnancy in patients in the ART program.

### 3.4. Identification of sncRNAs Associated with Implantation Potential of Embryo at Blastocyst Stage

Deep sequencing was performed on four samples of spent culture media from excellent blastocysts (4AA) on Day 5 that were unable to implant (*n* = 3 of 16) and able to implant (*n* = 1 of 15) after their transfer into the uterine cavity. Sequence reads aligned to miRBase v21 and piRNABase, with a count of at least five in one sample studied, were normalized in DeSeq2 package. The results of the comparison of deep sequencing data in the samples from blastocysts without the ability to implant (#4, #24, #34), with reference to that in the sample from blastocysts with the ability to implant (#8), in the form of the fold changes in the sncRNA expression level are presented in Table 5.

**Figure 7:**
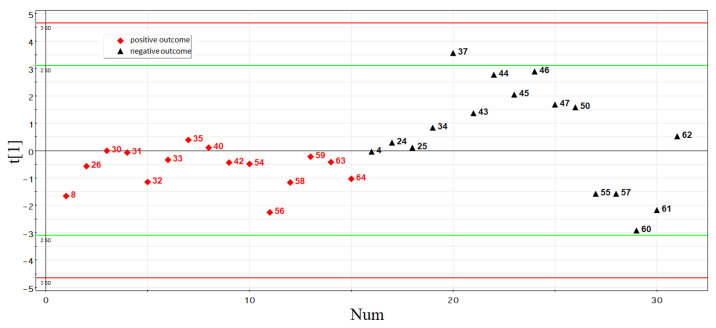
Principal component analysis (PCA) plot based on the sncRNA dataset for the blastocyst stage.

To validate the sequencing data by quantitative RT-PCR on the entire sample collection of the spent culture medium from blastocysts of good/excellent quality with different outcomes after transfer into the uterine cavity (*n* = 31), sncRNAs were selected, the expression level of which differed by at least a factor of two in at least two of the three spent culture medium samples from blastocysts without implantation relative to the spent culture medium samples from blastocysts with implantation (Table 5). PiRNA hsa_piR_020388 was chosen as the reference RNA due to its consistent expression level in all analyzed samples. The principal component analysis was used to determine the clustering of studied samples based on the logarithm to base two of fold change expression level of sncRNA in each of the spent culture medium samples relative to the culture medium without embryo, calculated as a (−ΔΔCt) value (−ΔΔCt data are presented in Table S1, Sheet 3). Figure 7 shows that spent culture medium samples from embryos with implantation potential (indicated by red squares) form a separate cluster and differ from most (62.5%) spent culture medium samples from embryos without implantation potential (indicated by black triangles) in the expression profile of sncRNAs. Samples #4, #25, #55, #55, #57, #60, and #61 from the blastocyst group without implantation were excluded from further analysis because of the similarity of their spent culture medium sncRNA expression profile with that of the spent culture medium from blastocysts with implantation. Patient #4 was diagnosed with secondary infertility for 4 years, two spontaneous miscarriages, and a separate diagnostic curettage for an endometrial polyp. Patient #25 was diagnosed with two years of secondary infertility, two ectopic pregnancies, one spontaneous miscarriage, and pelvic adhesions; she underwent a unilateral salpingectomy and a separate diagnostic curettage for an endometrial polyp. Patient #55 was diagnosed with four years of primary infertility, pelvic adhesions, and a separate diagnostic curettage for an endometrial polyp. Patient #57 was diagnosed with primary infertility for 7 years, external genital endometriosis with coagulation of endometriosis foci, separate diagnostic curettage, and one IVF/ICSI program without positive effect. Patient #60 was diagnosed with secondary infertility for 6 years, separate diagnostic curettage, first trimester uterine pregnancy termination, salpingectomy for ectopic pregnancy, adenomyosis, pelvic adhesions, and two IVF/ICSI programs without positive effect. Patient #61 was diagnosed with primary infertility for 2 years. Thus, the absence of pregnancy in this category of patients may be due to causes unrelated to embryo quality.

**Figure 8:**
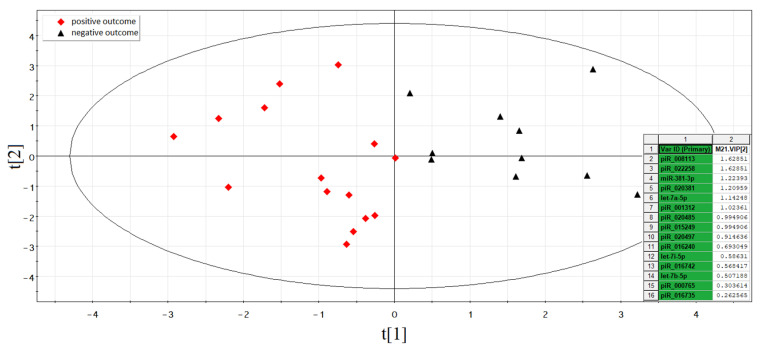
PLS-DA model for blastocyst stage.

According to the Partial Least Squares Discriminant Analysis (PLS-DA) model based on RT-PCR data without outlier samples, two clusters of data points can be clearly distinguished (Figure 8). The first one (highlighted in red) has an abscissa of less than 0.1 and represents the samples of spent culture medium from good/excellent blastocysts, implanted with the development of normal pregnancy after their transfer to the uterine cavity. The second cluster (highlighted in black) has an abscissa of more than 0.1 and represents the samples of spent culture medium from good/excellent blastocysts with no implantation.

The contribution of sncRNAs to the distribution of the data points on the score plot of the developed PLS-DA model was estimated by the Variable Importance in Projection (VIP) values for groups of embryos with and without implantation. The VIP values are presented as an insert in the right side of the score plot. The sncRNAs with VIP ≥ 1 and the highest impact for the differentiation of implanted from nonimplanted embryos were as follows: hsa_piR_008113 (VIP = 1.63), hsa_piR_022258 (VIP = 1.63), hsa-miR-381 (VIP = 1.22), hsa_piR_020381 (VIP = 1.21), hsa-let-7a-5p (VIP = 1.14), hsa_piR_001312 (VIP = 1.02), hsa_piR_020485 (VIP = 0.99), and hsa_piR_015249 (VIP = 0.99).

**Figure 9:**
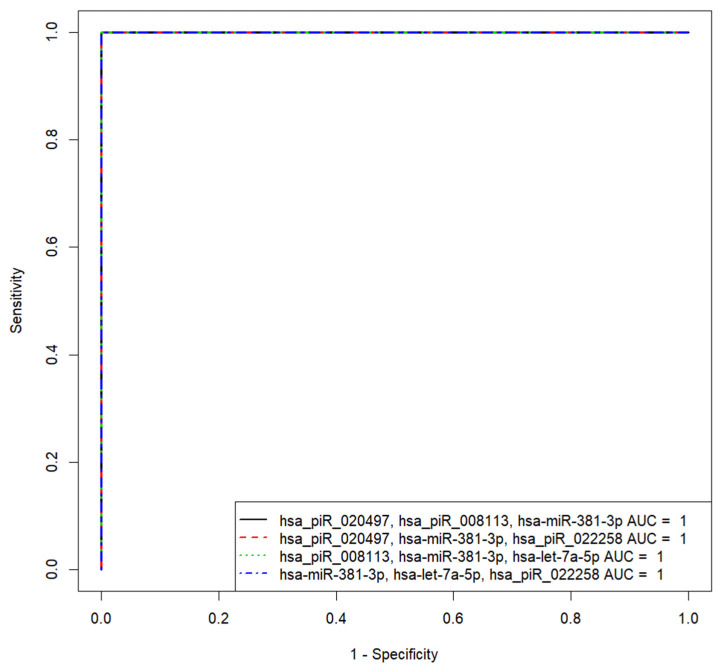
Receiver operating characteristic (ROC) curves of the logistic regression model 2 to predict implantation potential of embryo at the blastocyst stage.

Based on the (−ΔΔCt) values of sncRNA in the spent culture medium from blastocysts of couples with negative and positive results of the ART program, different variants of logistic regression model 2 for calculating the probability of the embryo implantation were developed (Figure 9). Receiver operating characteristic (ROC) curves of the developed logistic regression model 2 variants are presented in Table 6 and are described by formula 1 (see above). We found that the diagnostic accuracy of expression profiling of any combination of sncRNAs presented in Figure 8 was 100% (AUC = 1, sensitivity—100%, specificity—100%, true positive rate—1, false positive rate—0).

### 3.5. The Functional Significance of sncRNA Molecules Associated with the Implantation Potential of an Embryo at the Morula and Blastocyst Stages

**Figure 10:**
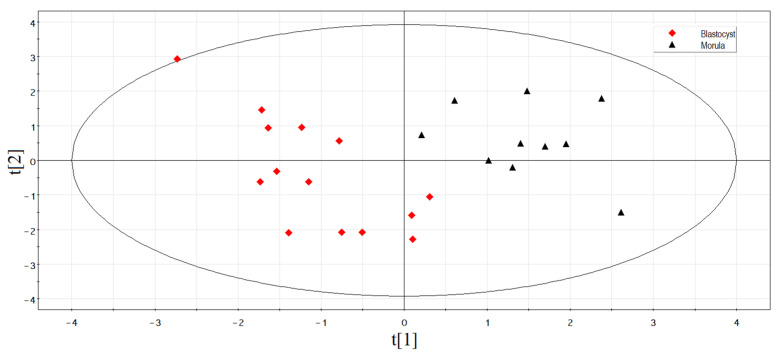
PLS-DA model for embryos at morula and blastocyst stages with implantation after transfer into the uterine cavity.

By comparing the expression profile of sncRNAs contributing to the implantation potential of embryos at the morula stage (Figure 4) and at the blastocyst stage (Figure 8), it was found that the same sncRNA molecules (hsa_piR_000765, hsa-let-7i-5p, hsa_piR_022258, hsa_piR_015249, hsa_piR_020485, hsa_piR_016735, hsa-let-7b-5p, and hsa_piR_020497) had different levels of expression at these two stages, which can be illustrated by the formation of two clusters of RT-PCR data according to the PLS-DA model (Figure 10). The first one (highlighted in red), with an abscissa of less than 0.25, and the second cluster (highlighted in black), with an abscissa of more than 0.25, represent the samples of spent culture medium from good/excellent blastocysts and morula, respectively, implanted with the development of normal pregnancy after their transfer to the uterine cavity. Such a clear division into two clusters indicates that the contribution of the same sncRNAs in determining the implantation potential of the embryo differs at different stages of its development. Moreover, for each analyzed stage of embryonic development, there is a unique list of sncRNAs contributing to the implantation potential of the embryo, namely, at the morula stage, hsa_piR_022628, hsa_piR_008112, hsa_piR_015026, hsa_piR_004695, hsa_piR_020381, and hsa_piR_019122, and at the blastocyst stage, hsa_piR_008113, hsa-miR-381-3p, hsa-let-7a-5p, hsa_piR_001312, and hsa_piR_016240.

**Figure 11:**
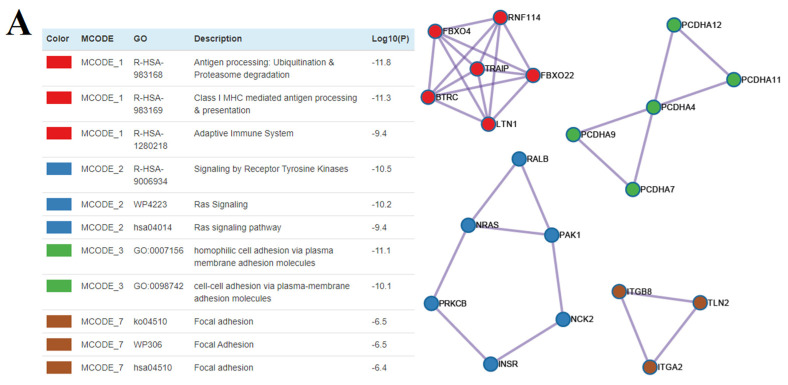

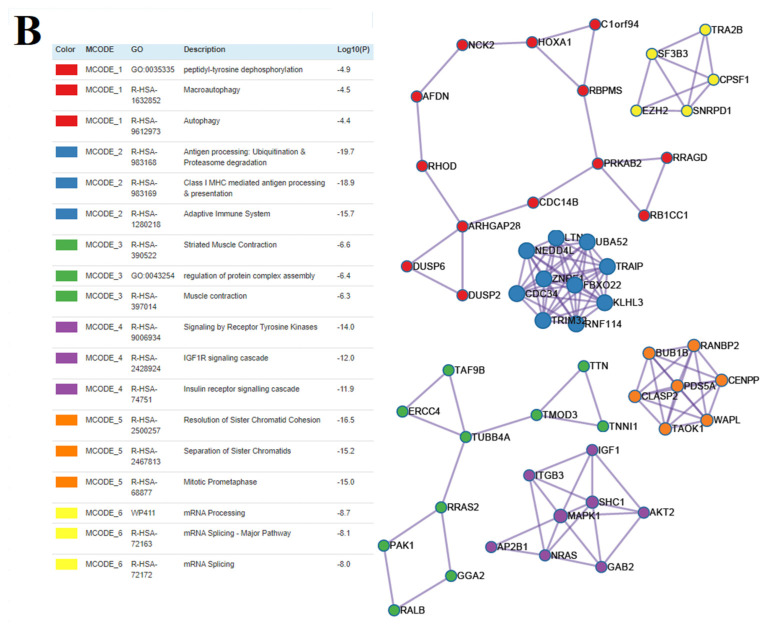
Network of proteins that form physical interactions with each other for morula (**A**) and blastocyst (**B**) stages while applying the Molecular Complex Detection (MCODE) algorithm. The full name of the proteins can be found in the NCBI database (https://www.ncbi.nlm.nih.gov/gene, last accessed on 15 June 2021) according to the RefSeq mRNA accession number indicated in Table S2 (Sheet 2).

In order to predict the possible targets of piRNAs, we used the GRCh38 database to download RefSeq transcript sequences (https://www.ncbi.nlm.nih.gov/genome/guide/human/, last accessed on 15 June 2021) and the miRanda algorithm with the alignment score of sc ≥ 170 and binding energy of en ≤ −20.0 kcal mol^−1^, as described in our recent manuscript [26]. The list of RNA targets for these piRNAs is presented in Table S2 (Sheet 1). RefSeq mRNA accessions were converted to gene symbols using the bioDBnet database (https://biodbnet-abcc.ncifcrf.gov/db/db2db.php, last accessed on 15 June 2021), and this information is presented in Table S2 (Sheet 2). The miRtargetlink database (https://ccb-web.cs.uni-saarland.de/mirtargetlink/, last accessed on 15 June 2021) was used to determine potential target mRNAs for hsa-let-7a-5p, hsa-let-7b-5p, hsa-let-7i-5p, and hsa-miR-381-3p, as presented in Table S2 (Sheet 3). sncRNA gene-targets, associated with implantation potential of the embryo at the morula and blastocyst stage, were compared to identify common and unique gene-targets for each of the stages (Table S2, Sheet 4). To assess the functional significance of the target genes, a Metascape enrichment analysis of protein coding by these genes was carried out using the Gene Ontology and Reactome databases. The functional relationships between the proteins as a subset of enriched terms are presented in Table S2, Sheet 5, for the morula stage and in Table S2, Sheet 6, for the blastocyst stage. While using BioGrid6, InWeb_IM7, and OmniPath8 databases, protein-protein interaction enrichment analysis was carried out in Metascape with the Molecular Complex Detection (MCODE) algorithm 9 application to identify densely connected network components, as shown in Figure 11A for the morula and in Figure 11B for the blastocyst stage.

Figure 11 clearly shows that among the proteins physically interacting with each other, there are those involved in the processes of ubiquitination and proteasome degradation at both the morula (MCODE_1, Figure 11A) and blastocyst stages (MCODE_2, Figure 11B), with a more numerous groups of these proteins in the latter. These proteins play a key role in the elimination of maternal factors after fertilization during the maternal-to-zygotic transition (MZT) and subsequent zygotic genome activation (ZGA) [35]. Another major eukaryotic protein degradation system in the embryo during preimplantation development is autophagy [36], and as it follows from Figure 11B (MCODE_1), 13 proteins with these functions physically interact and form a network at the blastocyst stage. While comparing Figure 11A,B, it is conspicuous that the proteins involved in cell adhesion (MCODE_3 and MCODE_7, Figure 11A) predominate at the morula stage, whereas most of the physically interacting protein networks at the blastocyst stage are involved in mRNA processing (MCODE_6, Figure 11B) and separation of sister chromatids during mitosis (MCODE_5, Figure 11B). Of note is the different protein composition of the networks involved in signaling through the receptor tyrosine kinases implicated in the regulation of a variety of biological responses, such as cell proliferation, migration, differentiation, and survival, at the morula (MCODE_2, Figure 11A) and blastocyst stage (MCODE_4, Figure 11B).

## 4. Discussion

In the present study, we identified key sncRNA molecules (miRNAs and piRNAs) that determine the implantation potential of the embryo at two consecutive stages of development, the morula and blastocyst. We found that the same sncRNA molecules (hsa_piR_000765, hsa-let-7i-5p, hsa_piR_022258, hsa_piR_015249, hsa_piR_020485, hsa_piR_016735, hsa-let-7b-5p, and hsa_piR_020497), contributing to the implantation potential of embryos, had different levels of expression at the morula and blastocyst stages. Moreover, for each analyzed stage of embryonic development, there was a unique list of sncRNAs contributing to the implantation potential of the embryo, namely, at the morula stage, hsa_piR_022628, hsa_piR_008112, hsa_piR_015026, hsa_piR_004695, hsa_piR_020381, and hsa_piR_019122, and at the blastocyst stage, hsa_piR_008113, hsa-miR-381-3p, hsa-let-7a-5p, hsa_piR_001312, and hsa_piR_016240. In this connection, we found 151 and 212 potential target proteins for sncRNA, specific for morula and blastocyst stages, respectively, as well as 148 sncRNA target proteins common to both stages (Table S2, Sheet 4). According to the Metascape enrichment analysis (Table S2, Sheets 5 and 6), these target proteins are implicated in different biological processes and signaling pathways, such as cell-cell junction organization, integrin-mediated cell adhesion, actin cytoskeleton organization (actin polymerization or depolymerization, actomyosin structure organization, actin-mediated cell contraction), microtubule polymerization or depolymerization, establishment or maintenance of cell polarity, mitotic sister chromatid segregation, embryonic morphogenesis, mRNA processing, ubiquitin-mediated proteolysis, autophagy, signaling pathways regulating pluripotency of stem cells, focal adhesion-PI3K-Akt-mTOR-signaling pathway, Hippo-signaling pathway, signaling by receptor tyrosine kinases, signaling by Rho GTPases, IGF1R-signaling cascade, signaling by EGFR, Wnt-signaling pathway, and signaling by FGFR.

It has been well established that the cytoskeleton, comprising actin filaments, microtubules and intermediate filaments, allows cells to transmit external signals to the internal biochemical signaling pathways, resulting in the gene expression and cellular behavior changes. Highly detailed research has been performed by Hui Yi Grace Lim and Nicolas Plachta [37], applying live imaging approaches to understand how the mammalian embryo forms and grows during the preimplantation stages of development. Beginning with the eight-cell stage, blastomeres polarize along their radial axis and undergo compaction through the reorganization, redistribution, and apical accumulation of microtubules, actin, and keratin intermediate filaments. The contact-free surface of each blastomere forms the apical domain, and the adhesion molecule E-cadherin is restricted to the basolateral cell-cell contacts. The main role in promoting embryo compaction during the 8-cell to 16-cell stage of development is the formation of filopodia at the apical cell cortex, whereas its retraction serves as a trigger for mitosis when cells decompact and become spherical. Compaction and embryo polarization are followed by the first spatial segregation of cells into two separate populations: apolar inner cells completely surrounded by the basolateral membrane and cell-cell contacts, and the cells on the outside of the embryo retaining an apical surface, which is devoid of cell-cell contacts and exposed to the exterior. The inner cells subsequently differentiate to form the primitive endoderm, which gives rise to extra-embryonic membranes, and the pluripotent epiblast, from which the entire embryo proper is derived. The cells on the outside of the embryo differentiate to form the trophectoderm, which gives rise to the placenta. Cell repositioning during the 16-cell stage is under the control of the Hippo-signaling pathway and downstream expression of fate-specifying markers that is critical for the correct development of the embryo [38]. In particular, both cell-cell adhesion and polarity act to regulate the junctional localization of Amot, a key member of the Hippo pathway required for the phosphorylation of Yap1. In the outer cells, Yap1 translocates into the nucleus to activate expression of trophectoderm-specific genes (transcription factors Gata2, Gata3, and Cdx2), whereas, in the inner cells, Yap1 is phosphorylated and therefore excluded from the nucleus. The specification of the trophectoderm and ICM lineages is accompanied by morphogenetic changes involving maturation of cell–cell junctions and generation of the blastocyst cavity. These processes again require rapid rearrangement and dynamic interactions between all three major cytoskeletal components. In addition, changes in the cytoskeleton are synchronized with those in the nucleoskeleton through the protein complex linker in the outer and inner nuclear membranes during multiple rounds of embryo cell divisions [39]. These connections provide a transmission signal from the plasma membrane to the nucleus, which can induce downstream changes in chromatin organization and embryo genome transcriptional activity.

**Figure 12:**
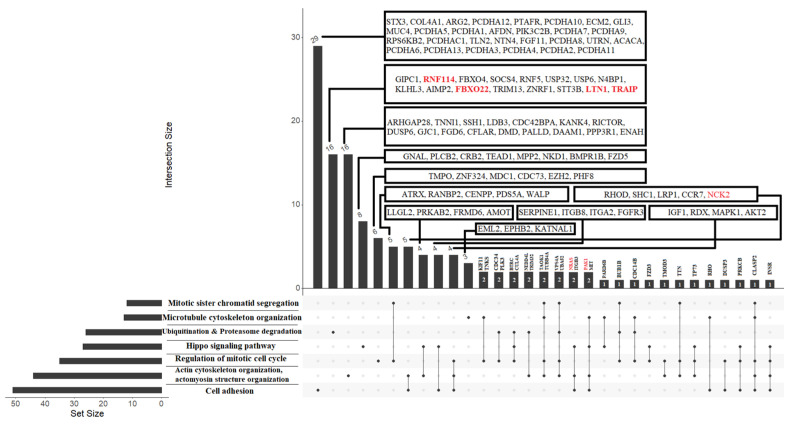
Functional significance of sncRNA target genes that determine the implantation potential of the embryo at the morula and blastocyst stages. The sncRNA target proteins common to the morula and blastocyst stages, which are components of the networks of physically interacting proteins in Figure 10A,B, are highlighted in red.

To our surprise, we found that the target genes of sncRNAs responsible for the implantation potential of the embryo at the morula and blastocyst stages are involved in the key biological processes in preimplantation embryogenesis as discussed above, namely cell adhesion, organization of actin cytoskeleton, microtubule cytoskeleton organization, regulation of mitotic cell cycle, mitotic sister chromatid segregation, Hippo-signaling pathway, as well as ubiquitination and proteasome degradation (Figure 12).

It is important to note that the protein products of several analyzed target genes are polyfunctional, i.e., they participate in more than one biological process indicated in Figure 12. Among them, CLIP-associated protein 2 (CLASP2) plays an essential role in epiblast cell differentiation into mesoderm and endoderm lineages during gastrulation [40]. Another protein from this group is PRKCB—protein kinase C, beta—which is involved in the regulation of mitochondrial integrity and oxidative phosphorylation [41]. Titin (TTN), a connectin, is megadalton-sized filamentous molecule that promotes human trophoblast invasiveness via the activation of the MAPK pathway, providing proper placentation and embryo development [42]. NRAS belongs to the family of Ras proteins, which control cell proliferation pathways, cell growth, and division [43]. Tropomodulin-3 (Tmod3) negatively regulates cell motility by controlling actin polymerization and stability, and in the case of Tmod3 knockdown, the gradual increase in the level of cytoplasmic actin and spindle migration are impaired during oocyte maturation [44]. CDC14B, a dual specificity phosphatase that counteracts cyclin-dependent kinase 1 (CDK1/CDC2A) action, is found to be a negative regulator of the 1-to-2-cell transition and of zygotic genome activation in mouse embryogenesis [45]. BUB1B (BUB1 mitotic checkpoint serine/threonine kinase B) has been reported to be associated with developmental potential of the human pre-implantation zygote [46]. The β3 integrin subunit (ITGB3) is implicated in the adhesion cascade for implantation, and being part of the integrin adhesion complexes such as ITGAV:ITGB3/SPP1 during implantation in humans, participates in the numerous intracellular pathways to regulate cell growth, proliferation, survival, gene expression, development, migration, and invasion [47,48]. Moreover, ITGAV:ITGB3 integrins define the window of implantation in women [49]. PAK1, a member of the p21-activated kinase (PAK) family of nonreceptor serine/threonine kinases, is involved in the assembly and disassembly of focal adhesions and stress fibers through myosin light-chain kinase phosphorylation; it plays important role in the Wnt-signaling pathway, phosphorylating β-catenin, followed by its nuclear translocation and activation of signal transduction cascades important in proliferation, morphogenesis, and differentiation. It can increase cell migration through activation of Paxillin and the MAPK/JNK pathway and can directly phosphorylate Snail to repress genes important for cell adherence and cell polarization [50]. It was shown that two other PAK family members, PAK2 and PAK4, play crucial roles in the development of the embryo, as their gene deletion results in embryonic lethality in mice. NCK2 interacts with the PAK family and their upstream activators to regulate cytoskeletal dynamics [51]. Jacquet K. and colleagues found that Nck2−/− cells delay in cytokinesis (the final stage of cell division), display longer intercellular bridges, and often fail to complete abscission [52]. As for TUBB4, it is the crucial element of the microtubules participating in the turnover of focal adhesions, which regulate cell polarization and migration, and is responsible for efficient transport of N-cadherin to the cell membrane [53]. Depletion of VPS4, AAA ATPase, alters centrosome numbers and size and causes defects in polar spindle formation and chromosome segregation during cell division [54]. Finally, it is important to note the regulator of Hippo-signaling pathway TAOK1/3, identified as direct kinases for LATS1/2 [55], which in turn acts together with phosphorylated Amot to enhance the phosphorylation and nuclear exclusion of Yap1, allows the expression of pluripotency-associated genes in the inner cells of the 16-cell embryo [37].

In the present study, we found that some of the protein products of the sncRNA target genes responsible for embryo implantation potential are characterized by direct physical protein-protein interactions, with the formation of protein networks (Figure 11A,B), among which are those involved in the processes of ubiquitination and proteasome degradation at both the morula (MCODE1, Figure 11A) and blastocyst stages (MCODE2, Figure 11B). These proteins play a key role in the elimination of maternal factors after fertilization during the maternal-to-zygotic transition (MZT) and subsequent stages of embryogenesis, ensuring timely selective turnover of proteins and inhibition or promotion of protein activity at a certain stage of embryonic development [35]. Among them are proteins specific to the morula stage (FBXO4, BTRC), specific to the blastocyst stage (UBA52, NEDD4L, ZNRF1, CDC34, TRIM32, KLHL3), and common to the morula and blastocyst stages (LTN1, TRAIP, FBXO22, RNF114). FBXO4 is the E3 ubiquitin ligase with a proven role to promote ubiquitination and degradation of apoptosis suppressor Mcl-1 [56], cyclin D1 [57], and intercellular cell adhesion molecule-1 (ICAM-1) [58], therefore regulating cell survival, cell division, and cell-cell contacts. BTRC (b-transducing repeat containing) protein, as a part of the E3 ligase complex, promotes ubiquitination of phosphorylated b-catenin, which participates in the adherens junctions, providing an anchor between the cytoskeleton and cadherins, and plays a role as a transcriptional coactivator of the expression of a growing number of target genes [59]. Ubiquitin A-52 residue ribosomal protein fusion product 1 (Uba52), being the major source of ubiquitin protein for covalent modification of proteins in the proteasome system, is essential in early embryogenesis, enabling cell cycle progressing, mitosis, RNA translation and processing, protein catabolism, and chromatin remodeling [60]. In the case of the Uba52 gene modified embryos using the CRISPR/Cas9 gene editing tool, delay in embryo development, abnormal blastomere nuclear structure, and decreased percentage of blastocyst formation were revealed. NEDD4L, an E3 ubiquitin ligase, has been shown to negatively regulate the Wnt signaling responsible for cell proliferation, polarity, and fate determination during embryonic development [61]. The relationship between the ubiquitination system and mitosis is carried out by the protein ubiquitin-conjugating enzyme Cdc34, which participates in the degradation of the protein kinase Wee1—a key negative regulator of mitosis, and this proteolysis event is required for a timely entrance into mitosis [62]. Among participants in ubiquitin-mediated protein degradation, common for morula and blastocyst stages, RNF114 (Ring finger 114) protein was identified as a key molecule for clearance of maternal factors to support maternal-to-zygotic transition and subsequent zygotic genome activation [35]; FBXO22, an F-box receptor subunit of SCF E3 ligase, is a key regulator of cell growth, cell cycle, and cell migration through ubiquitylation of CD147, PTEN, p21, LKB1, KLF4, Bach1, Snail, and HDM2 [63]; the ubiquitin ligase Ltn1, the major component of the ribosome-associated protein quality control system, detects and eliminates aberrant polypeptides through their ubiquitination and degradation by the proteasome [64]; and TRAIP, a ubiquitously expressed nucleolar E3 ubiquitin ligase, is crucial in accurate chromosome segregation during mitosis [65].

Due to the existence of networks in which proteins with different biological functions physically interact with each other, changes in the molar concentration of one of the network components will result in the disruption of the entire network. Since all proteins that form these networks through physical interaction are potentially regulated by sncRNAs that determine the implantation potential of the embryo at the morula or blastocyst stage, an imbalance in the expression level of these sncRNAs can lead to disruption of the physical interaction of the corresponding proteins and thus disrupt the biological processes that lead to the formation of high-quality embryos.

Thus, the implantation potential of the embryo at the morula or blastocyst stage is determined by the expression level of stage-specific sncRNAs, which regulate the expression level of target proteins. The precise coordination of these processes in space and time establishes the proper development of the embryo.

## 5. Conclusions

Two logistic regression models were developed to predict the implantation potential of the embryo with 100% sensitivity and 100% specificity, based on the expression profile of sncRNAs in the embryo spent culture medium at the morula stage (model 1: various combinations of hsa_piR_022258, hsa-let-7i-5p, hsa_piR_000765, hsa_piR_015249, hsa_piR_019122, and hsa_piR_008112) and blastocyst stage (model 2: various combinations of hsa_piR_020497, hsa_piR_008113, hsa-miR-381-3p, hsa_piR_022258, and hsa-let-7a-5p). Protein products of sncRNA potential target genes, associated with the ability of the embryo to implant, participate in the selective turnover of proteins through the ubiquitination system at a certain stage of embryonic development, in the organization of the structure of various cell cytoskeleton systems interacting with both adhesion proteins and nuclear chromatin, and in regulation of the activity of the Hippo-signaling pathway, which determines the fate specification of the blastomers.

## Figures and Tables

**Table 1:** sncRNA sequence data.

sncRNA ^1^	Accession Number ^1^	Nucleotide Sequence of Sense Primer for PCR, 5′-3′	PCR Primers Annealing Temperature, °C
hsa_piR_001312	DQ571813	attggtggttcagtggtagaattctcgcctg	46.2
hsa_piR_002769	DQ573726	tcataagtgggagctaaatgatgagaac	46.2
hsa_piR_008113	DQ581032	tgaggtagtaggttgtatagttttagggtc	46.2
hsa_piR_016240	DQ592148	tgttaaccgaaaggttggtggttcgagcc	48.9
hsa_piR_016742	DQ593049	ccggctagctcagtcggtagagcatgaga	48.9
hsa_piR_019122	DQ596252	gacagagaaaacaaggtggtgaactatgccc	46.2
hsa_piR_020365	DQ597975	ggccgtgatcgtatagtggttagtactctg	46.2
hsa_piR_000765	DQ570956	agcattggtggttcagtggtagaattctcgc	49
hsa_piR_000807	DQ571005	agcctgatgatgtcctcctccagttgccgc	53
hsa_piR_004695	DQ576442	tcctggaccagcctgatgatgtcctcctcc	57
hsa_piR_004880	DQ576715	tccttgtcctggaccagcctgatgatgtcct	45
hsa_piR_008112	DQ581031	tgaggtagtagattgtatagttgtggggtagt	46.2
hsa_piR_015026	DQ590548	tggttcagtggtagaattctcgcctcc	45
hsa_piR_015249	DQ590830	tgtagtcgtggccgagtggttaaggc	46.2
hsa_piR_015462	DQ591122	tgtcctgggccagcctgatgatgtcctcctc	45
hsa_piR_016735	DQ593039	ccgcctgggaataccgggtgctgtaggctta	50
hsa_piR_017716	DQ594453	ttccctggtggtctagtggttaggattcggc	45
hsa_piR_020381	DQ597997	ggcgggagtaactatgactctcttaaggta	53
hsa_piR_020485	DQ598159	ggggatgtagctcagtggtagagcgcatgct	53
hsa_piR_020497	DQ598177	tgtagctcagtggtagagcgcgtgct	45
hsa_piR_022258	DQ600471	tactacctgattggtcgggtgtgagc	48.9
hsa_piR_022628	DQ600952	tagagcatgagactcttaatctcagggtcgtg	48.9
hsa_piR_020388	DQ598008	ggctcgttggtctaggggtatgattctcgg	45
hsa-miR-381-3p	MIMAT0000736	tatacaagggcaagctctctgt	52.7
hsa-let-7a-5p	MIMAT0000062	Hs_let-7a_2 miScript Primer Assay, MS00031220	55
hsa-let-7b-5p	MIMAT0000063	Hs_let-7b_1 miScript Primer Assay, MS00003122	55
hsa-let-7i-5p	MIMAT0000415	Hs_let-7i_1 miScript Primer Assay, MS00003157	55

^1^ piRNAbank (http://pirnabank.ibab.ac.in/cgi-bin/accession.cgi, last accessed on 15 June 2021) for piRNAs; miRBase (http://www.mirbase.org/search.shtml, last accessed on 15 June 2021) for miRNAs.

**Table 2:** Comparative characteristics of clinical data and parameters of the IVF/ICSI protocol in couples, depending on the outcome of the ART program.

Clinical Data and Parameters of the IVF/ICSI Protocol	Negative Outcome (*n* = 85)	Positive Outcome (*n* = 25)	Mann–Whitney Test, *p* ^1^
	Me (Q1;Q3) ^1^		
Age of Female	32.5 (30;35)	31 (30;32.75)	0.112915
Menarche, age	13 (13;14)	13 (13;14)	0.941626
Duration of menstrual bleeding, days	5 (4;5)	4 (4;5)	0.195459
The length of the menstrual cycle, days	28 (28;30)	28 (28;29.75)	0.256809
Prolactin (120.0–500.0, mIU/L)	301.2 (198.55;352.75)	284.35 (194.25;337.57)	0.473241
17-OH-progesterone (0.3–3.0, nmole/L)	1.58 (1.1;1.96)	1.3 (1.22;1.65)	0.538915
Dehydroepiandrosterone sulphate (0.9–11.7, µmole/L)	5.2 (3.1;7.6)	3.45 (2.45;6.8)	0.283792
Cortisol (200.0–550.0, nmole/L)	315 (245.05;380.5)	306.15 (259.55;373.3)	0.901587
Testosterone (0.5–2.5, nmole/L)	1.61 (1.12;2)	1.15 (0.74;1.67)	0.017549
Anti-müllerian hormone (1.0–10.6, ng/mL)	2.21 (1.52;3.1)	3.05 (2.35;4.33)	0.007737
Thyroid-stimulating hormone (0.4–3.5, mIU/L)	1.77 (1.15;2.49)	1.27 (0.98;2.14)	0.182973
Free thyroxine (10.0–25.0, pmole/L)	15.1 (12.22;18)	14.75 (12.06;18.3)	0.950685
Luteinizing hormone (2.3–15.0, IU/L)	5.9 (4.75;7.08)	4.8 (4.15;5.74)	0.036986
Progesterone, luteal phase of the menstrual cycle (16.0–95.0, nmole/L)	27.6 (19.02;43)	31.58 (24.05;47)	0.251808
Follicle-stimulating hormone (2.0–10.0, IU/L)	7.55 (6.53;8.95)	6.41 (6.18;8.3)	0.094989
Estradiol (150.0–450.0, pmole/L)	154.5 (100.05;206.01)	165.75 (145.25;230)	0.32861
Gonadotropin Dosage, IU	1725 (1293.75;1950)	1425 (1143.75;1762.5)	0.103318
Stimulation Duration, days	9 (8;10)	9 (8.25;10)	0.406869
Number of follicles on the day of administration of the trigger of the final oocyte maturation	8.5 (5;11)	8.5 (5.25;10)	0.986806
Number of OCCs	7 (4.25;12)	8 (4.25;10.75)	0.820127
Number of Metaphase II (MII) Oocytes	6 (3;9.75)	6 (4;7.75)	0.842485
Age of Male	34 (32;38)	33 (31;34.75)	0.112239
Sperm Concentration, Million per Milliliter	60 (31.25;107)	61.5 (41;91)	0.944129
Morphologically Normal Spermatozoa, %	2 (2;3)	2 (1.25;3)	0.582895
Sperm with Progressive Motility, %	56 (44;67.75)	60 (37.5;67)	0.904815
Number of two pronuclei embryos	5 (3;8)	5.5 (3.25;7.5)	0.765231
Blastocyst number	1 (1;3)	1 (1;2.75)	0.606454
Duration of infertility	4 (2;6)	3 (2;5)	0.302846
Number of IVF attempts in history	0 (0;1)	0 (0;0.75)	0.189145

^1^ Data are presented as a median (Me) and quartiles Q1 and Q3 in the Me format (Q1; Q3), with an indication of the statistical significance (*p*) using the Mann–Whitney test.

**Table 3:** Small noncoding RNA deep-sequencing data for spent culture medium from morula.

sncRNA	Spent Culture Medium from Morula without Potential for Implantation ^1^				Spent Culture Medium from Morula with Potential for Implantation ^1^			Culture Medium without Embryo ^1^	Mann–Whitney Test (*p*-Value)
	#179	#167	#193	#173	#150	#118	#154	#207	
hsa_piR_022628	7	21	63	21	77	119	133	14	0.00484937
hsa_piR_015249	75	160	135	200	240	245	310	210	0.009863152
hsa_piR_016735	23	107	171	124	205	232	265	204	0.011349613
hsa_piR_004880	139	277	572	291	616	651	839	549	0.012957723
hsa_piR_004695	139	280	576	295	622	651	844	551	0.013187947
hsa_piR_015462	278	560	1152	590	1244	1302	1688	1102	0.013187947
hsa_piR_000807	278	560	1152	590	1244	1300	1684	1102	0.01323007
hsa_piR_022258	0	0	0	0	10	68	68	0	0.014900377
hsa_piR_008112	2	0	8	2	9	7	9	0	0.026879189
hsa_piR_019122	4	5	24	13	28	23	54	35	0.030210112
hsa-let-7i-5p	39	2	0	25	151	23	101	18	0.036138954
hsa_piR_020365	9	15	12	27	19	53	40	9	0.036254562
has-let-7b-5p	37	2	7	33	160	29	95	17	0.037297033
hsa_piR_015026	20	60	65	55	90	65	140	15	0.039937759
hsa_piR_020381	1122	6942	5946	7146	9432	7413	14,019	5532	0.042809887
hsa_piR_000765	540	432	300	565	1146	1248	4014	240	0.043505506
hsa_piR_020497	170	122	189	140	246	165	223	296	0.045522073
hsa_piR_002769	0	0	0	0	0	82	164	0	0.04660816
hsa_piR_020388	80	41	64	53	86	100	54	83	0.115425375
hsa_piR_020485	483	210	546	357	364	308	413	532	0.349984126

^1^ Normalized sncRNA read count.

**Table 4:** Parameters of the logistic regression model 1.

Biomarker	AUC	Sp, %	Se, %	Cutoff	i	K
“hsa_piR_022258, hsa-let-7i-5p, hsa_piR_000765”	1	100	100	0.5	67.9	−1248.05 −1127.34 −834.25
“hsa_piR_015249, hsa_piR_022258, hsa-let-7i-5p, hsa_piR_000765”	1	100	100	0.5	−15.05	63.06 −211.33 −178.26 −146.78
“hsa_piR_019122, hsa_piR_022258, hsa_piR_008112, hsa_piR_000765”	1	100	100	0.5	−280.49	−26.33 −191.08 295.17 −107.57
“hsa_piR_019122, hsa_piR_022258, hsa-let-7i-5p, hsa_piR_000765”	1	100	100	0.5	12.02	31.37 −154.67 −92.71 −99.38

**Table 5:** SncRNA deep sequencing data for spent culture medium from blastocysts.

sncRNA	sncRNA Read Count				Fold Changes in the sncRNA Expression Level		
	#4	#34	#24	#8	#4/#8	#34/#8	#24/#8
hsa-let-7a-5p	3835	443	1541	7783	0.493	0.057	0.198
hsa-let-7b-5p	20737	254	1834	26297	0.789	0.010	0.070
hsa-let-7i-5p	3465	90	1055	4072	0.851	0.022	0.259
hsa-miR-381-3p	120	1	83	475	0.253	0.002	0.175
hsa_piR_008113	173	153	93	652	0.265	0.235	0.143
hsa_piR_001312	486	486	549	2151	0.226	0.226	0.255
hsa_piR_000765	8850	7248	2004	15162	0.584	0.478	0.132
hsa_piR_016735	26	118	81	347	0.075	0.340	0.233
hsa_piR_015249	175	625	580	280	0.625	2.232	2.071
hsa_piR_020497	163	596	562	181	0.901	3.293	3.105
hsa_piR_016240	54	126	108	1	54.000	126.000	108.000
hsa_piR_016742	65	120	150	30	2.167	4.000	5.000
hsa_piR_020485	2429	1841	1876	952	2.551	1.934	1.971
hsa_piR_020388	448	499	546	386	1.161	1.293	1.415

**Table 6:** Parameters of the logistic regression model 2.

Biomarker	AUC	Sp, %	Se, %	Cutoff	i	K
«hsa_piR_020497, hsa_piR_008113, hsa-miR-381-3p»	1	100	100	0.5	1860.371618	−1999.768215, 3345.749357, −98.52044368
«hsa_piR_020497, hsa-miR-381-3p, hsa_piR_022258»	1	100	100	0.5	1860.371618	−1999.768215, −98.52044368, −3345.749357
«hsa_piR_008113, hsa-miR-381-3p, hsa-let-7a-5p»	1	100	100	0.5	−6233.293319	6761.631563, −516.0692614, −369.5848655
«hsa-miR-381-3p, hsa-let-7a-5p, hsa_piR_022258»	1	100	100	0.5	−6233.293319	−516.0692614, −369.5848655, −6761.631563

## Supplementary Materials

The following are available online at https://www.mdpi.com/article/10.3390/life11121328/s1, Table S1, Sheet 1: The parameters of the logistic regression models to predict pregnancy based on the hormone profile in the peripheral blood of the patients, Table S1, Sheet 2: Quantitative RT-PCR data (−∆∆Ct values) of sncRNA expression level in the morula spent culture medium samples, Table S1, Sheet 3: Quantitative RT-PCR data (−∆∆Ct values) of sncRNA expression level in the blastocyst spent culture medium samples, Table S1, Sheet 4: The parameters of the logistic regression models to predict implantation potential of embryo at the blastocyst stage based on the expression level of sncRNAs associated with implantation potential of embryo at the morula stage, Table S2: Potential gene-targets of sncRNAs implicated in the implantation of the embryo at morula and blastocyst stages.
